# Ni loaded SnS_2_ hexagonal nanosheets for photocatalytic hydrogen generation *via* water splitting†

**DOI:** 10.1039/d2ra07954b

**Published:** 2023-01-16

**Authors:** Niteen Jawale, Sudhir Arbuj, Govind Umarji, Manish Shinde, Bharat Kale, Sunit Rane

**Affiliations:** a Centre for Materials for Electronics Technology (C-MET) Off Pashan Road, Panchawati Pune-411008 Maharashtra India sudhir1305@gmail.com sunit@cmet.gov.in +912025898180 +912025899273

## Abstract

Herein we have prepared the Ni-decorated SnS_2_ nanosheets with varying concentrations of Ni from 1 to 10 mol% (1, 2.5, 5, and 10 mol%) and studied their various physicochemical and photocatalytic properties. The chemical reduction technique was utilized to load the Ni nanoparticles on SnS_2_ nanosheets. The synthesized Ni decorated SnS_2_ (denoted as Ni-SnS_2_) was characterized using different spectroscopic techniques such as X-ray diffraction, diffuse reflectance UV-vis and photoluminescence spectroscopy, field emission scanning electron microscopy (FESEM), and field emission transmission electron microscopy (FETEM). XRD revealed the formation of the highly crystalline hexagonal phase of SnS_2_ but for nickel loading there is no additional peak observed. Further, the as-prepared Ni-SnS_2_ nano-photocatalyst shows absorption behaviour in the visible region, and photoluminescence spectra of the Ni-SnS_2_ nanostructures show band edge emission centred at 524 nm, and the peak intensity decreases with Ni loading. The FE-SEM and FE-TEM confirm the formation of hexagonal sheets having evenly distributed Ni nanoparticles of size ∼5–10 nm. BET surface area analysis was observed to be enhanced with Ni loading. The photocatalytic performance of the prepared Ni-SnS_2_ nanosheets was evaluated for hydrogen generation *via* water splitting under a 400 W mercury vapour lamp. Among the prepared Ni-SnS_2_ nanostructures, the Ni loaded with 2.5 mol% provided the highest hydrogen production *i.e.*, 1429.2 μmol 0.1 g^−1^ (% AQE 2.32) in four hours, almost 1.6 times that of pristine SnS_2_*i.e.*, 846 μmol 0.1 g^−1^. Furthermore, the photocatalytic performance of the catalyst is also correlated with the photoconductivity by measuring the photocurrent. The photoconductivity of the samples is revealed to be stable and the conductivity of 2.5 mol% Ni-SnS_2_ is higher *i.e.* 20 times that of other Ni-SnS_2_ and pristine SnS_2_ catalysts.

## Abstract 2

Schematic of photocatalytic H_2_ generation using Ni/SnS_2_ nanosheets along with the energy levels of SnS_2_ and Ni nanostructures.

## Graphical abstract

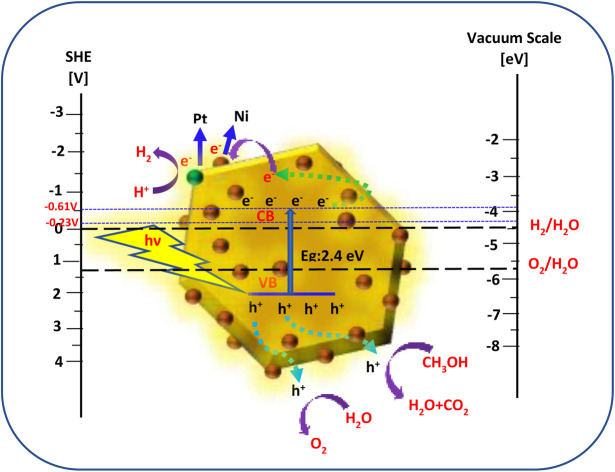

## 1 Introduction

In the world of science and technology, the increasing energy demand and rapid depletion of fossil fuels are becoming major issues in the present scenario.^1,2^ So, the need for the development of alternate clean, renewable energy sources is an area that has received significant attention from the scientific community. Sunlight and water are abundantly available in nature and can be utilized to produce H_2_*via* water splitting.^3–6^

Solar energy is one of the cleanest sources of energy. Many different technologies such as photovoltaic cells and photochemical water splitting can be used to harness solar energy. It is believed that the photo-assisted water-splitting reaction on the semiconductor surface is one of the most promising technologies for hydrogen production and provides direct conversion of sunlight into chemical fuels.^7–10^ In 1972 the pioneering report by Fujishima and Honda on photoelectrochemical (PEC) water splitting on a TiO_2_ photo-electrode attracted wide and continual research interest for the development of environmentally friendly, and stable photocatalysts.^11–13^ This photocatalytic water splitting using nanostructured semiconductor offers a low-cost eco-friendly approach for sustainable hydrogen production.

The nanostructured semiconductors' metal oxides and chalcogenides are playing a vital role in the photocatalysis technique. For having higher photocatalytic performance, the semiconductor catalyst must have an optimum bandgap, optimum energy positions, and chemical as well as biologically stable nature.^14–20^ Over the last two-three decades, the use of nanostructured semiconductor materials especially TiO_2_, ZnO, SnO_2_, ZnS, SnS_2_, MoS_2_, CdS, CeO_2_, Ta_2_O_5_, Nb_2_O_5_, SrTiO_3_, *etc.* has been used as a potential photocatalyst.^21–45^ However, the conversion efficiency of photon energy into H_2_ of the studied photocatalytic materials is limited by the factors such as poor absorption ability of the visible light, fast recombination of photoexcited charge carriers, low surface catalytic reaction efficiency, poor stability, *etc.* Since from a few decades, researchers have proposed many different strategies or routes to overcome these restrictions, including co-catalyst loading, crystal facet engineering, construction of different surface morphology with porous structure, band gap engineering, and construction of homo or hetero-junctions (binary, ternary), *etc.*^46–60^ To avoid the recombination of charge carriers, noble metal nanoparticles have been widely used as efficient co-catalysts decorated on semiconductor photocatalysts. Many researchers have reported that noble metal NPs, such as Au, Pt, Ag, Rh, Ni-Pt, Pd, Cu-Pd, and Au-Pd have been reported resulting in enhanced overall photocatalytic activity by enhancing the electron–hole charge separation.^61–71^

In this regard, SnS_2_ nanostructures were synthesized by hydrothermal method and were *in situ* decorated with Ni as a co-catalyst having different concentrations of Ni *i.e.*, 1 mol%, 2.5 mol%, 5 mol%, and 10 mol% through a simple thermal reduction method. The detailed physico-chemical characterisation and influence of Ni loading on SnS_2_ towards its photocatalytic performance were investigated.

## 2 Experimental section

### 2.1 Materials

Stannic(iv) chloride (SnCl_4_·5H_2_O) was purchased from Otto Chemicals Pvt. Ltd, thiourea (CH_4_N_2_S) from Qualigens fine chemicals, ethylenediamine (EDA) (C_2_H_8_N_2_), nickel chloride from Fischer Scientific, ethylene glycol (EG) Merck Life Science Pvt Ltd, and hydrazine hydrate were purchased from Sisco Research Laboratories Pvt Ltd. All chemicals were used such as without further purification.

### 2.2 Synthesis of tin disulphide

The SnS_2_ hexagonal nanosheets were synthesized as per the previously reported method.^33^ In a typical experimental procedure, 10 mmol (3.5 g) of stannic chloride was dissolved in 30 ml of distilled water in a beaker, while 40 mmol (3.04 g) of thiourea were dissolved in 30 ml of distilled water in another beaker and stirred for 10–20 min at room temperature. Thiourea solution was then added dropwise into stannic chloride solution with constant stirring for 10 min. After that, 7.5 mmol (0.5 ml) of EDA was added to the solution with constant stirring for 5 minutes. Then the transparent reaction mass was transferred into a Teflon-lined stainless-steel autoclave, sealed, and heated at 200 °C for 24 h. Upon further completion of the reaction, the autoclave was naturally cooled down to room temperature, and a yellow product was obtained. The obtained sample was rinsed several times with distilled water followed by ethanol and collected by centrifugation and then dried at 60 °C in a vacuum oven and used for further characterizations.

### 2.3 Ni nanoparticles loading on SnS_2_ nanosheets

The Ni-loaded SnS_2_ nanosheets (Ni-SnS_2_) were prepared as follows: for 1 mol% loading firstly, 0.053 g of nickel chloride and hydrazine hydrate 0.53 ml was dissolved in 60 ml EG in a 250 ml beaker and stirred for 30 min. Second, as prepared SnS_2_ nanosheets were suspended into the suspension and stirred for 30 min to disperse the nanoparticles. Then 1 M aqueous NaOH solution was added dropwise into the suspension and stirred for 60 min at 60 °C. The obtained product was washed several times with distilled water followed by ethanol wash and collected by centrifugation and then dried at 60 °C in a vacuum oven for 24 hours. The same reaction was followed for loading of Ni with 2.5, 5 and 10 mol% respectively.

### 2.4 Materials characterization

Powder X-ray diffraction (XRD) analysis of the as-prepared samples was carried out using Bruker AXS D8 Advanced model equipped with a monochromator and Ni-filtered Cu Kα radiation (wavelength = 1.5418 Å) in the 2*θ* range of 10° to 70° with the scan rate of 1° min^−1^. Diffuse reflectance UV-visible absorbance spectra (DRS) were recorded at room temperature in the range of 200–800 nm using the Shimadzu UV-vis-NIR spectrophotometer (Model UV-3600). The morphology of the as-synthesized samples was observed using a field-emission scanning electron microscope (FESEM, HITACHI S-4800). The high-resolution TEM (HRTEM) and selected area electron diffraction (SAED) were also recorded using JEOL JEM-200FS FETEM. The BET surface area of the prepared SnS_2_ and Ni-SnS_2_ nanostructures were calculated from the N_2_ adsorption isotherms at liquid N_2_ temperature using the Quantachrome NOVATouch LX1 instrument. The sample was degassed in a vacuum at 120 °C for 2 h prior to the BET analysis.

### 2.5 Photocatalytic activity measurement

The photocatalytic activity of the prepared nanostructures was carried out by observing H_2_ generation *via* water splitting.

#### 2.5.1 H_2_ generation

The photocatalytic hydrogen evolution *via* water splitting over prepared SnS_2_ and Ni-SnS_2_ nanostructures was performed as per the previously reported method.^23^ The cylindrical quartz vessel reactor (100 ml size) was used for photocatalytic H_2_ generation. The system was sealed with a septum arrangement to remove the evolved gas through a gas-tight syringe for quantifying the amount of gases evolved. The mercury vapour lamp (400 W) fitted in a quartz condenser having water circulation arrangement in order to absorb the *iR* radiation to minimize the heating effect was used as a light source. For photocatalytic activity measurements, 20 mg of the catalyst with 1 wt% preloaded platinum as a co-catalyst was dispersed into 25 ml distilled water and 5 ml methanol solution. Further, 0.1 M Na_2_S and 0.1 M Na_2_SO_3_ were added as a sacrificial agent in the above solution. In order to remove the dissolved gases, nitrogen gas was purged through this mixture for 30 min. After purging the nitrogen gas through the reaction vessel, it is kept under the mercury vapor lamp, and analyzed the evolved gases after an interval of one hour, using a gas chromatograph (Shimadzu: Model GC 2014) equipped with 5 Å molecular sieves column. The amount of hydrogen generation was calculated according to the fitted standard curve.

#### 2.5.2 Photoconductivity measurement

Additionally, we have investigated the photoconductivity of synthesized SnS_2_ and Ni-SnS_2_ nanostructures. For photoconductivity measurement, SnS_2_ and Ni-SnS_2_ powder films were deposited on a pre-patterned silver inter-digited electrode printed on 96% alumina (substrate) with an inter-electrode spacing of 100 μm and a total path length of 25 mm. The deposited electrodes were directly used for *I*–*V* measurement. The measurements were performed using a Kelvin probe connected to spring-loaded pressure contacts at room temperature. The entire setup was maintained in the metallic chamber (shield) in order to reduce the electrical noise effect. *I*–*V* measurements were carried out using Keithley 4200 semiconductor characterization system (SCS) integrated with a photo-emission system (1000 W Xenon lamp and 1.5 AM (air-mass) ratio).

## 3 Results and discussion

### 3.1 X-ray diffraction analysis

**Fig. 1:**
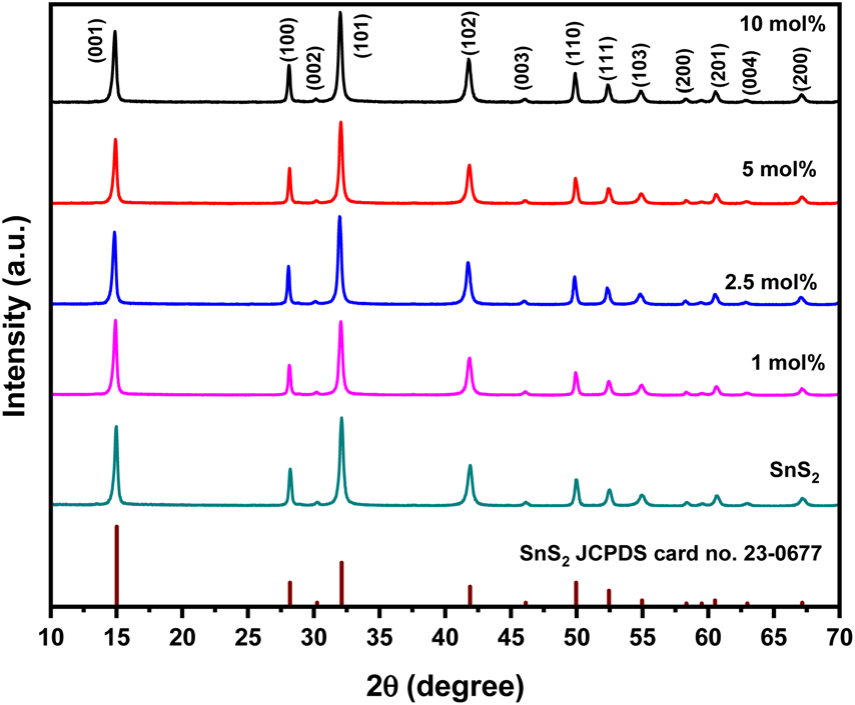
XRD pattern of pure SnS_2_, 1 mol% Ni-SnS_2_, 2.5 mol% Ni-SnS_2_, 5 mol% Ni-SnS_2_ and 10 mol% Ni-SnS_2_.

In order to reveal the crystal structure of the as-synthesized SnS_2_ and Ni-SnS_2_ nanostructures, an X-ray diffraction analysis was conducted and depicted in Fig. 1.

It can be seen that the XRD pattern exhibits diffraction peaks at 2*θ* = 15, 28.1, 30.2, 32.1, 41.8, 46.1, 49.9, 52.4, 54.9, 58.3, 60.6, 62.9, 67.1, 70.3 and 77.4° coincide with (001), (100), (002), (101), (102), (003), (110), (111), (103), (200), (201), (004), and (202) planes of pure SnS_2_ having a hexagonal crystal structure, respectively. The peaks in the XRD patterns shows the formation of the hexagonal phase of SnS_2_ (JCPDS card no. 23-0677). Because of the high crystallinity of SnS_2_ nanosheets, no distinct diffraction peaks of Ni can be found in the XRD patterns of the as-synthesized Ni-SnS_2_ composites. XRD pattern of pristine and recycled 2.5 mol% Ni-SnS_2_ is also appeared to be same indicating the stability of catalyst even after three recycles (Fig. S1 ESI†). However, the existence of metallic Ni in the as-synthesized Ni-SnS_2_ composites was confirmed by XPS analysis.

### 3.2 UV-visible diffuse reflectance spectroscopy (UV-DRS) analysis

**Fig. 2:**
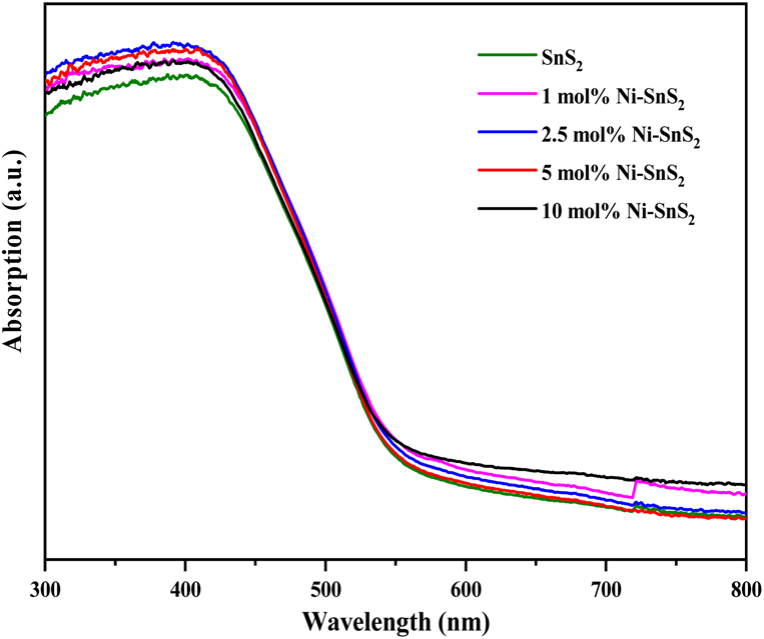
UV-DRS Spectra of pure SnS_2_, 1 mol% Ni-SnS_2_, 2.5 mol% Ni-SnS_2_, 5 mol% Ni-SnS_2_ and 10 mol% Ni-SnS_2_.

**Fig. 3:**
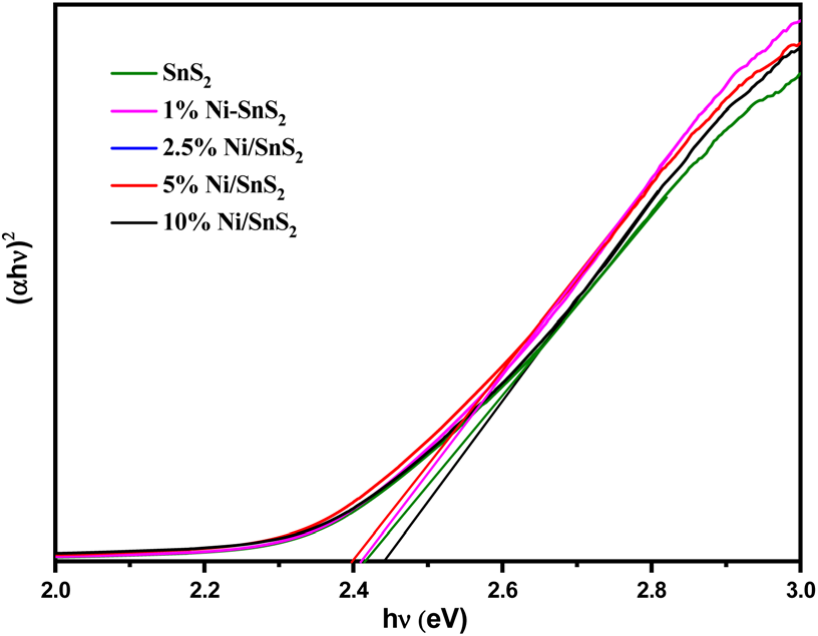
Tauc's spectra of pure SnS_2_, 1 mol% Ni-SnS_2_, 2.5 mol% Ni-SnS_2_, 5 mol% Ni-SnS_2_ and 10 mol% Ni-SnS_2_.

UV-vis-DRS of the as-synthesized pristine SnS_2_ and Ni-SnS_2_ with different Ni concentrations (depicted in Fig. 2) were measured to evaluate their band gap as well as photo-absorption behaviour. The pristine SnS_2_ and different concentrations of Ni-SnS_2_ both show an absorbance edge around 550 nm corresponding to the optical band gap of 2.4 eV, also confirmed by the Tauc's plot (shown in Fig. 3). There was no noticeable change observed in the absorption edge with the increment in nickel content. This indicates that nickel was just deposited on the surface instead of being fused into the lattice structure of SnS_2_.

### 3.3 Photoluminescence study

**Fig. 4:**
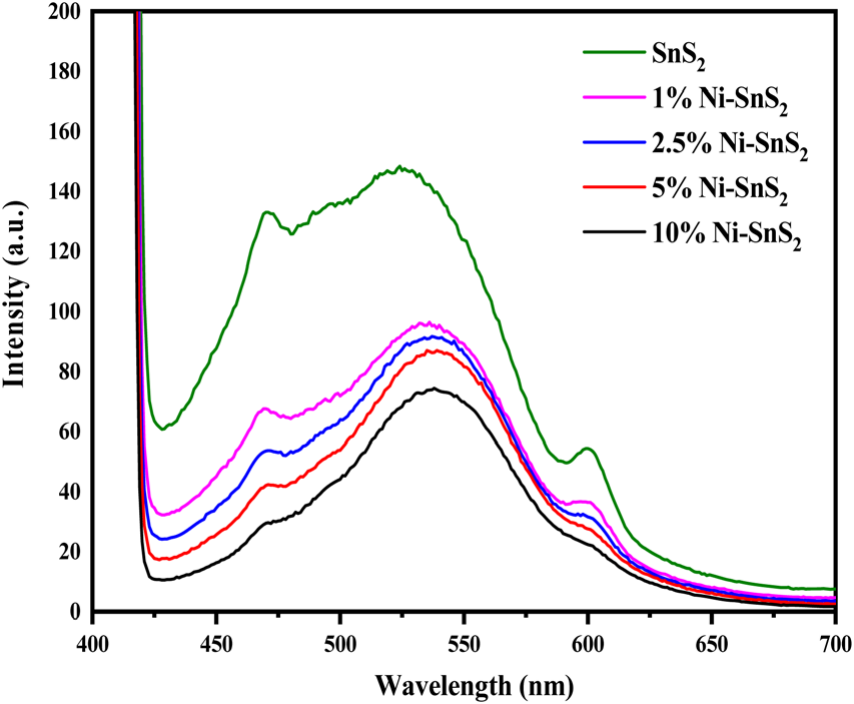
Photoluminescence spectra of pure SnS_2_, 1 mol% Ni-SnS_2_, 2.5 mol% Ni-SnS_2_, 5 mol% Ni-SnS_2_ and 10 mol% Ni-SnS_2_.

The PL spectra of synthesized SnS_2_ and Ni-loaded SnS_2_ nanosheets having different Ni concentrations were obtained with an excitation wavelength of 350 nm and are shown in Fig. 4. The PL spectra of SnS_2_ as well as Ni-SnS_2_ nanosheets, show strong emission peaks centred at ∼525 nm. The band edge emission for SnS_2_ was observed to be around 525 nm and this peak shifts to 540 nm with increasing Ni loading. Additionally, as evident from the PL graph, the intensity of the emission peak of pristine SnS_2_ and Ni-SnS_2_ decreases with increment in Ni concentration and it is lowest for the sample with 10 mol% loading of Ni. This slaking might be due to the presence of Ni nanoparticles on the surface of SnS_2_ nanosheets and confirms the transfer of photogenerated electrons from the conduction band of SnS_2_ to Ni nanoparticles.

### 3.4 X-ray photoelectron study

**Fig. 5:**
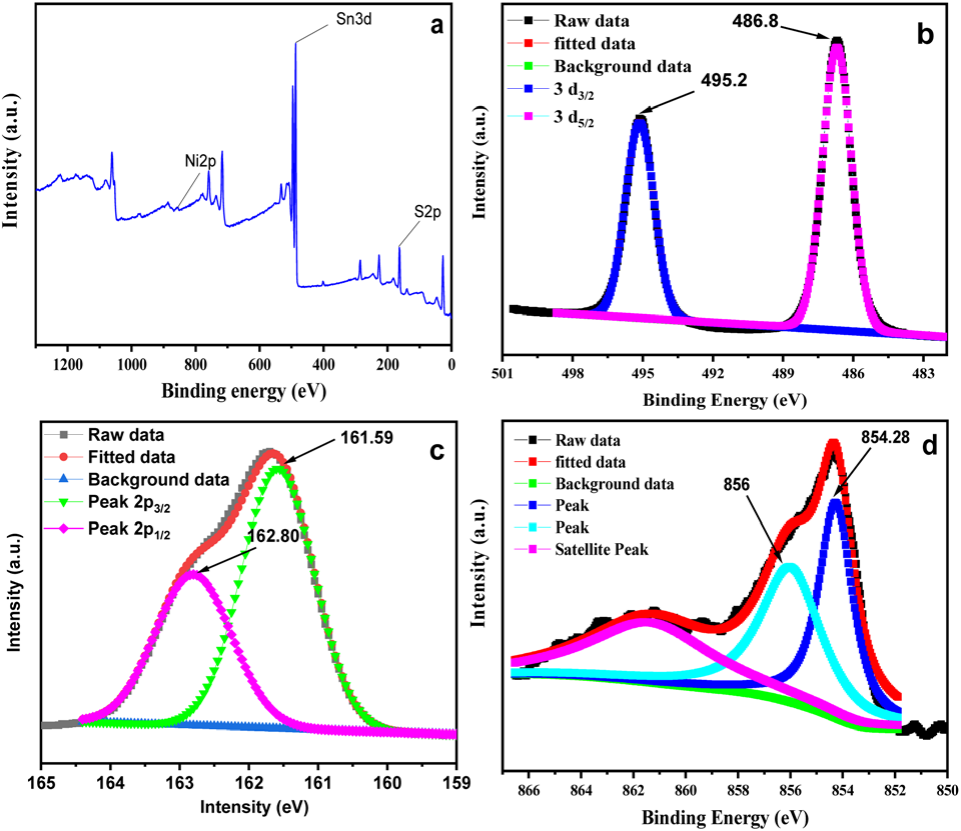
(a) Survey XPS spectrum of Ni-SnS_2_, (b) high-resolution XPS spectra of Sn 3d, (c) S 2p, (d) Ni 2p.

The XPS survey spectrum depicts in Fig. 5a reveals that the as-synthesized 2.5 mol% Ni-SnS_2_ nanostructures consist of Sn, S and Ni. Fig. 5b–d represent the high-resolution deconvoluted XPS spectrum of Sn 3d, S 2p and Ni 2P for Ni-SnS_2_ nanostructures. Further the XPS of pristine SnS_2_ is also depicted in Fig. S2 (ESI†). The high-resolution Sn 3d core-level spectrum shows two prominent peaks at binding energies of 486.73 and 495.15 eV corresponding to the Sn 3d_5/2_ and Sn 3d_3/2_ levels respectively. The high-resolution spectrum of S depicts in Fig. 5c contains peaks at 161.59 and 162.80 eV binding energy that corresponds, respectively, to S 2p_3/2_ and S 2p_1/2_ levels. The binding energy values observed for Sn 3d and S 2p spectra are in good agreement with Sn^4+^ and S^2−^ of SnS_2_. Additionally, the major peaks with binding energies of 856 and 854.28 eV are corresponding to Ni^3+^ as well as Ni^2+^ respectively (Fig. 5d). It has been observed that over the time metallic Ni get oxidized by losing electron and may easily generates high valence state Ni *viz.*, +2 and +3. In the prepared nanostructures the presence of Ni along with Ni oxide on SnS_2_ were confirmed using XPS analysis.^72^

### 3.5 Morphological and nano-structural analysis

#### 3.5.1 FE-SEM analysis of pure SnS_2_ and Ni-SnS_2_

**Fig. 6:**
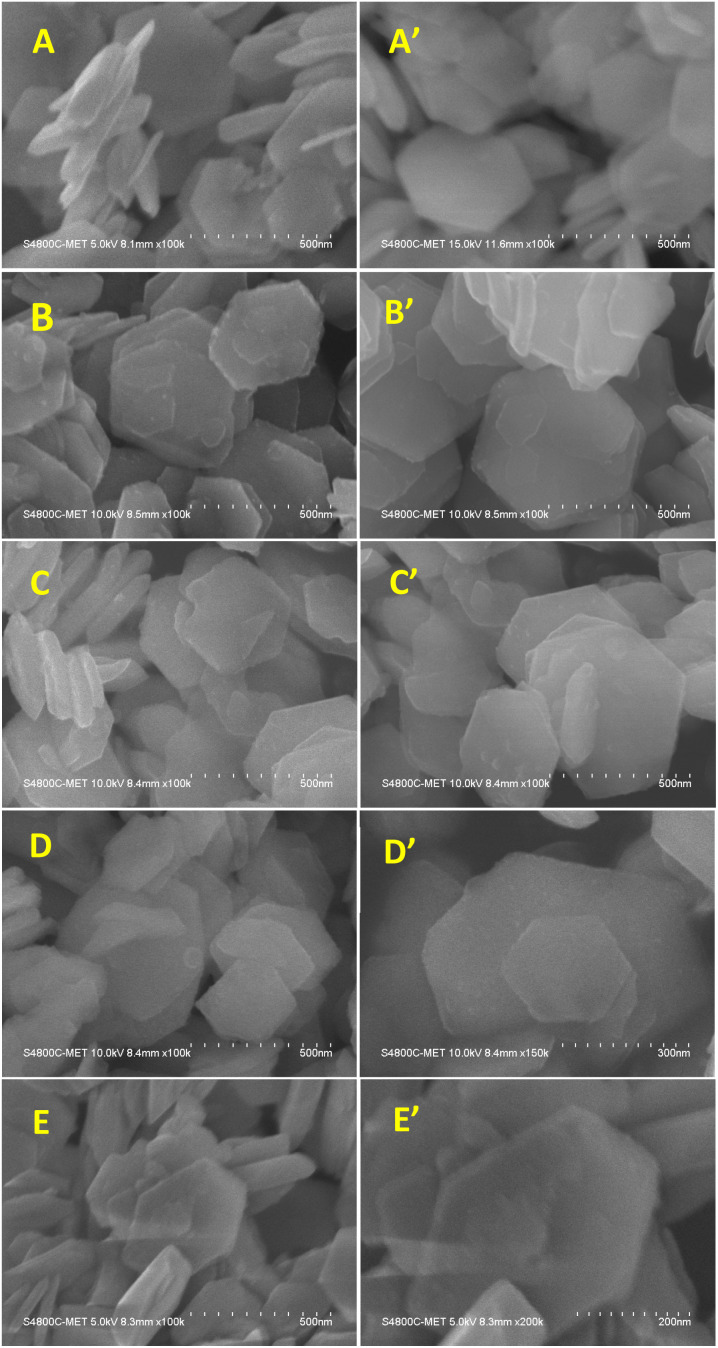
FESEM images of SnS_2_ and Ni-SnS_2_ nanosheets, (A and A′) pure SnS_2_ nanosheets, (B and B′) 1 mol% Ni-SnS_2_, (C and C′) 2.5 mol% Ni-SnS_2_, (D and D′) 5 mol% Ni-SnS_2_, (E and E′) 10 mol% Ni-SnS_2_.

FE-SEM micrographs of synthesized SnS_2_ and Ni-SnS_2_ nanostructures are shown in Fig. 6. The FE-SEM image indicates the formation of two-dimensional hexagonal nanosheets having size in the range of 400 to 600 nm with thickness of 20 to 30 nm. The EDA is used as the capping agent to form SnS_2_ nanostructure in such a way that, it results in the formation of plate-like structure and directing two-dimensional growths. The Ni loading with varying concentrations was deposited on SnS_2_ surface by the thermal reduction method. The FE-SEM of Ni-loaded SnS_2_ also shows the same morphology as pure SnS_2_ and some spherical particles were observed on the surface of SnS_2_ sheets that might be of Ni. As the mole percentage of Ni increases the small spherical nanoparticles of Ni are observed on SnS_2_ nanosheets. At the maximum loading of 10 mol% of Ni-loaded SnS_2_ nanosheets, a chunk of Ni nanoparticles is observed on the surface of SnS_2_. Overall, the Ni nanoparticles are uniformly distributed over the SnS_2_ surface.

#### 3.5.2 FE-TEM analysis of pure SnS_2_ and Ni-SnS_2_

**Fig. 7:**
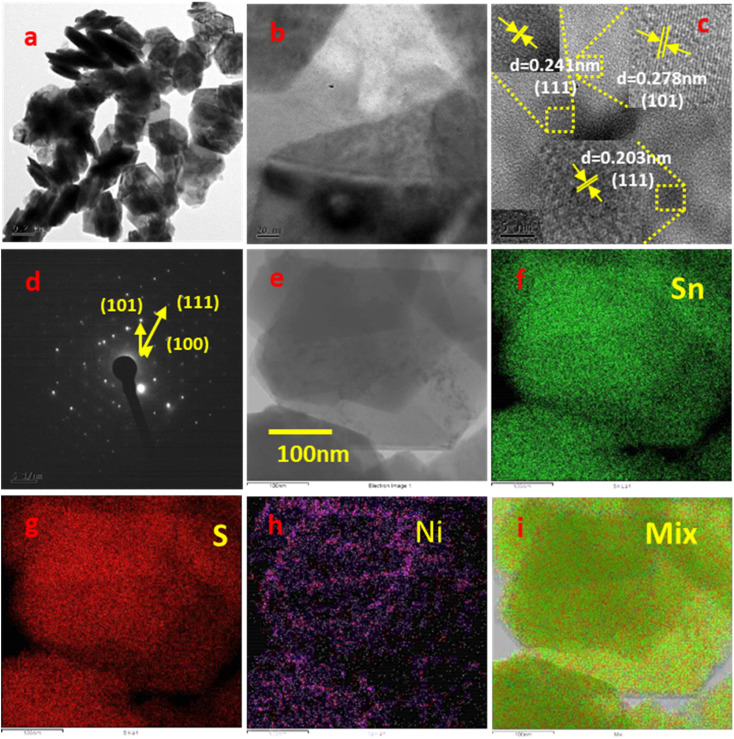
FETEM images of 2.5 mol% Ni-SnS_2_; (a, b and e) low magnification images, (c) HR-TEM image, (d) SAED pattern, (e–i) elemental mapping images of Ni-SnS_2_.

**Fig. 8:**
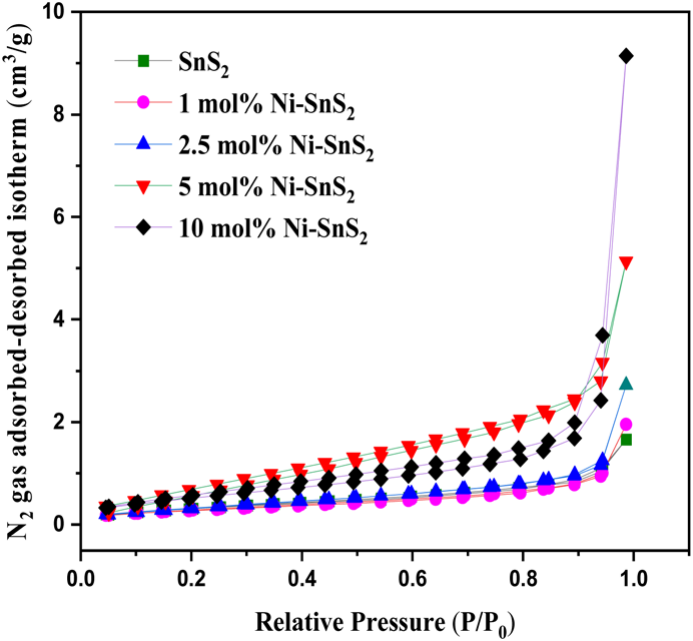
BET N_2_ gas adsorbed–desorbed isotherm curve of sample SnS_2_ and Ni-SnS_2_.

The orientation and distribution of Ni nanoparticles on the surface of SnS_2_ nanosheets were observed by FE-TEM analysis of a 2.5 mol% Ni-SnS_2_ sample shown in Fig. 7. The Fig. 7a and b shows the formation of uniformly distributed hexagonal shaped plates having the size in the range 300–500 nm along with homogeneous distribution of smaller Ni and NiO nanoparticles on the surface of SnS_2_ nanosheets. The HR-TEM image shown in Fig. 8c exhibits the interplanar spacing of *d* = 0.278 nm, 0.203 nm and 0.241 nm corresponds to (101), (111) and (111) *hkl* planes of SnS_2_, Ni and NiO nanostructures respectively. The size of Ni and NiO nanoparticles was observed as 5–10 nm. The HR-TEM analysis clearly indicates the presence of cubic Ni along with NiO nanoparticles uniformly distributed over hexagonal SnS_2_ surface. The Ni nanoparticles may get oxidised to form the NiO. The SEAD (selective area electron diffraction) with a hexagonal phase of SnS_2_. The diffraction spots can be indexed as (101) (111) and (100) planes confirming the main exposed facets of hexagonal SnS_2_. TEM associated elemental mapping analysis further confirmed the even distribution of Sn, and S within Ni-SnS_2_ (Fig. 7e–g). Further, the presence of Ni nanoparticles is displayed by the corresponding elemental mapping image as shown in Fig. 7h, while the overall distribution of Ni, Sn and S is substantiated in their overlap elemental mapping (Fig. 7i). Thus, FE-TEM analysis validates the XRD, and FESEM results and confirmed the formation of highly crystalline 2D SnS_2_ nanoplates with a homogeneous distribution of Ni and NiO nanoparticles on the surface of SnS_2_ nanosheets. Further the FETEM EDAX analysis for the 2.5 mol% Ni-SnS_2_ shows the loading of 2.18 wt% Ni over the SnS_2_ (Fig. S6†).

#### 3.5.3 BET surface area analyser

**Fig. 9:**
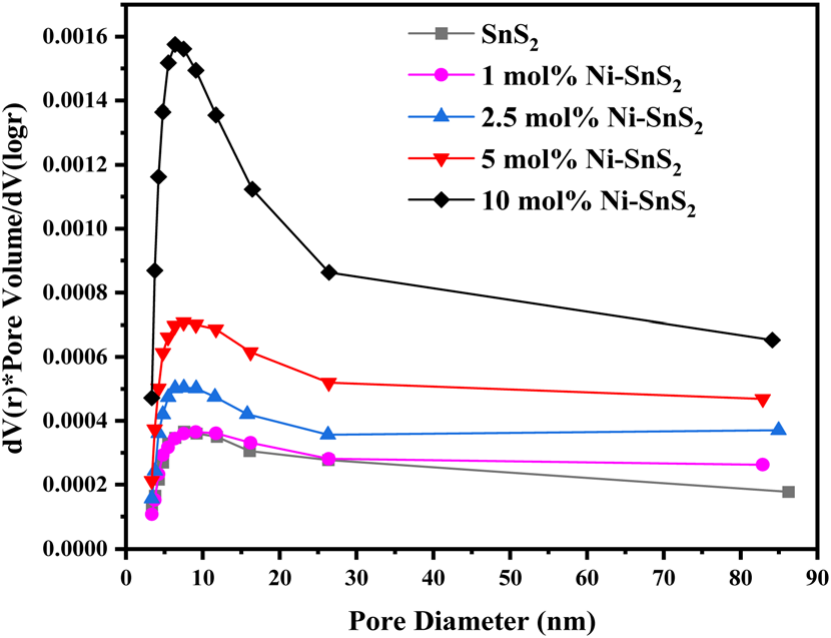
BJH pore size distribution curves of pristine SnS_2_, 1 mol% Ni-SnS_2_, 2.5 mol% Ni-SnS_2_, 5 mol% Ni-SnS_2_, 10 mol% Ni-SnS_2_.

The BET surface area and the porosity of the prepared SnS_2_ and Ni-SnS_2_ were investigated. Fig. 8 shows the nitrogen adsorption–desorption isotherm curves of pristine SnS_2_ and Ni-SnS_2_ nanostructures. The BET surface area of SnS_2_ and 1 mol% Ni-SnS_2_, 2.5 mol% Ni-SnS_2_, 5 mol% Ni-SnS_2_, 10 mol% Ni-SnS_2_ is observed as 7.8, 8.1, 10.2, 14.1, 18.4 m^2^ g^−1^ respectively. Therefore, as the Ni concentration on the surface of SnS_2_ nanosheets increases, the surface area of the photocatalyst also increases, this indicates that the loading of Ni nanoparticles is beneficial for the rise of the surface area of the catalyst. The pore size of the SnS_2_ and Ni-SnS_2_ nanostructure (Fig. 9) showed different levels of pores. The SnS_2_ and Ni-SnS_2_ nanostructures had a pore diameter less than 10 nm with a maximum diameter of ∼6 nm indicating that are mesoporous in nature.

### 3.6 Photocatalytic activity measurements

**Fig. 10:**
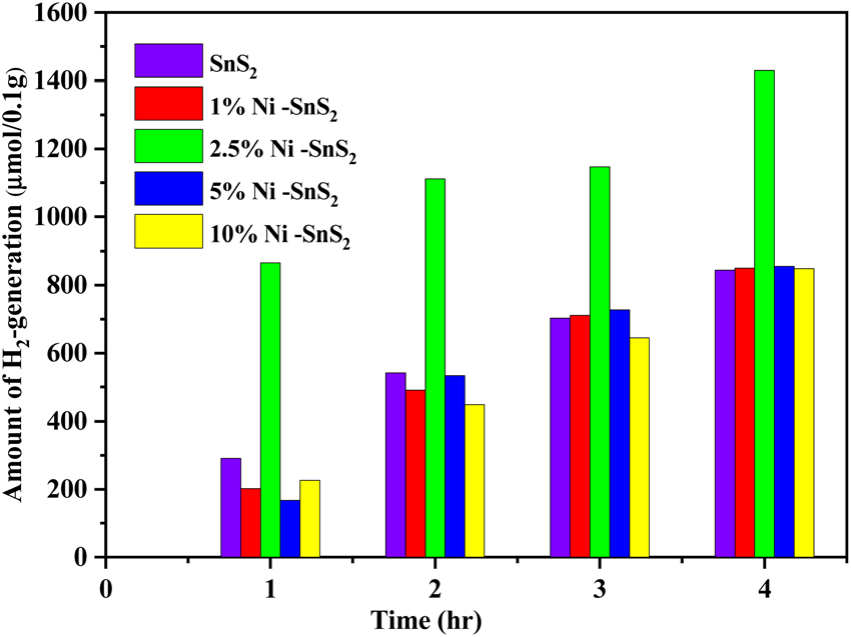
Photocatalytic H_2_ generation using pure SnS_2_, 1 mol% Ni-SnS_2_; 2.5 mol% Ni-SnS_2_, 5 mol% Ni-SnS_2_ and 10 mol% Ni-SnS_2_.

The investigation of photocatalytic H_2_ generation occurred by as-synthesized SnS_2_ and Ni-SnS_2_ nanosheets with different concentrations of Ni loading are depicted in Fig. 10. The 2.5 mol% Ni-SnS_2_ showed the highest H_2_ generation around 1429.2 μmol 0.1 g^−1^ within four hours of irradiation times. The Ni-SnS_2_ with Ni amounts of 1 mol%, 5 mol%, 10 mol%, and pristine SnS_2_ generates 850.09 μmol, 855.45 μmol, 848.67 μmol, and 843.75 μmol of hydrogen gas per 0.1 g of catalysts respectively shown in Table 1. The 2.5 mol% of Ni-SnS_2_ concentration showed almost 1.68 times higher photocatalytic activity as compared to pristine SnS_2_. Among the other concentrations of Ni-SnS_2_, the 2.5 mol% Ni-SnS_2_ shows the highest photocatalytic activity due to the generation of a large number of electron–hole pairs and its effective electron–hole pair separation. As nickel nanoparticles concentration increases more than 2.5 mol% loading, the Ni nanoparticles covers the effective surface of SnS_2_ nanosheets results in a shading effect and results in lowering the photocatalytic activity. For comparison of photocatalytic activity, the % apparent quantum efficiency (AQE) is also calculated (Table 1) and found to be around 2.32% for the 2.5 mol% Ni-SnS_2_. To support the effective charge generation, we have carried out the photoconductivity measurements of all the prepared catalysts.

**Table 1:** Surface area, H_2_-generation, apparent quantum efficiency using prepared SnS_2_, Ni-SnS_2_ nanostructures

S. no.	Sample code	BET surface area (m^2^ g^−1^)	H_2_ generation (μmol 0.1 g^−1^) in 4 h	% [AQE]^a^
1	SnS_2_	7.8	843.75	1.36
2	1 mol% Ni-SnS_2_	8.1	850.09	1.37
3	2.5 mol% Ni-SnS_2_	10.2	1429.2	2.32
4	5 mol% Ni-SnS_2_	14.1	855.45	1.38
5	10 mol% Ni-SnS_2_	18.4	848.67	1.37

a The % AQE calculated using the H_2_ generation values for 0.1 g catalyst per h.^75^

The recycling study of the catalyst is an important parameter to find out the stability and reusability of the catalyst. For this purpose, the Ni-SnS_2_ that exhibits higher photocatalytic activity (2.5 mol% Ni-SnS_2_) was recycled three times for H_2_ generation. After each cycle, the catalyst was separated from the suspension by centrifugation, washed with deionized water and ethanol, dried and weighted for the next cycle. Fig. S5† shows the recycling performance for the 2.5 mol% Ni-SnS_2_ nanoplates in photocatalytic H_2_ generation. During the recycling study the catalyst shows some loss of catalytic performance for third cycle, this might be due to the loss of catalyst during the washing process. After third recycle, the amount of H_2_ generated is around 1160.12 μmol of H_2_, 0.1 g^−1^ in 4 h of irradiation of time. This was clearly indicating that the synthesized Ni-SnS_2_ have good photocatalytic stability. There is no change observed in surface morphology confirmed by the FESEM and FE-TEM analysis (Fig. S3 and S4†) of 2.5 mol% Ni-SnS_2_ nanoplates before and after water splitting reaction under 400 W mercury vapor lamp for three cycles respectively.

### 3.7 Mechanism of photocatalytic H_2_ generation of prepared Ni-SnS_2_ nanostructures

**Fig. 11:**
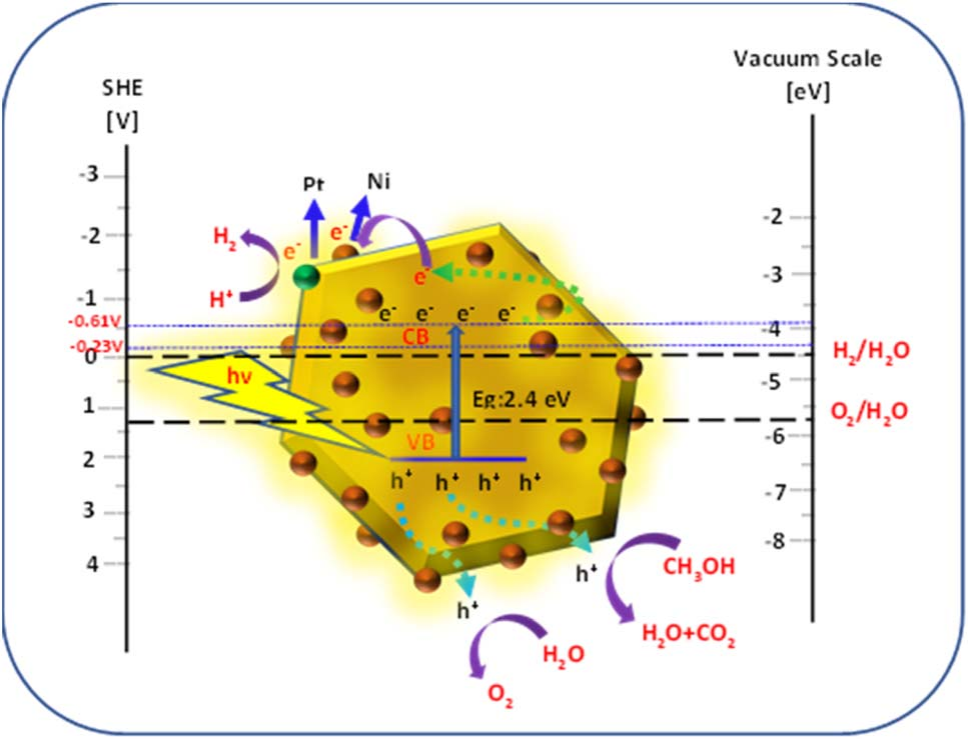
Schematic of band energy levels of Ni-SnS_2_ nanostructures for photocatalytic H_2_ generation/relative to the redox potential of water.^73,74^

The schematic of the mechanism of photocatalytic H_2_ generation is shown in Fig. 11. Upon irradiation of the Ni-SnS_2_ nanostructures by light, electrons are excited from the valence band (VB) to the conduction band (CB), leaving holes in the valence band. The photoexcited electrons and holes are migrated to the surface of these semiconductors, further, the electrons in the conduction band initiate the reduction of H^+^ to generate hydrogen and the holes oxidizes water to generate oxygen.

For H_2_ evolution, the CB potential of the catalyst must be more negative than the reduction potential of water. Providing an easy pathway for photogenerated electrons and holes for migrating towards reaction sites present on the surface. This decreases the recombination probability and enhances photocatalytic efficiency. In the present case of Ni-SnS_2_, improved activity with Ni loading may be due to the effective electron accepter property of Ni. The CB level of SnS_2_ is −0.61 V above the H_2_ evolution potentials. The photoexcited electrons from CB of SnS_2_ can migrate to Ni nanoparticles due to potential gradient and then react with H^+^ ions produced by oxidation of water to form H_2_. The Ni is having more electron affinity and its energy level is just −0.23 V more negative than the H_2_ evolution potential. Due to this, it easily captures the photogenerated electrons and is used further for H_2_O reduction. Further, Ni on SnS_2_ nanosheets can form the Schottky barrier and facilitates electron capture for H_2_ generation. Beyond the optimized loading, the activity was found to be decreased.

### 3.8 Photoconductivity measurement

**Fig. 12:**
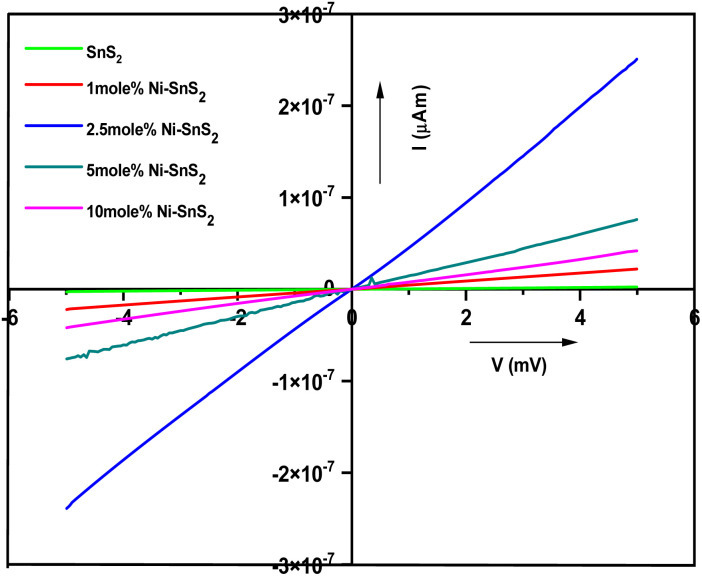
Current–voltage characteristics of pristine SnS_2_ and Ni-SnS_2_ nanosheets (1 mol% Ni-SnS_2_; 2.5 mol% Ni-SnS_2_, 5 mol% Ni-SnS_2_ and 10 mol% Ni-SnS_2_).

Photoconductivity measurements of pure SnS_2_ and Ni-SnS_2_ nanosheets were performed in air at room temperature with a bias voltage from −5 V to +5 V under the illumination of solar simulator light to record the photo-induced effect. The *I*–*V* characteristics for pure SnS_2_ and Ni-SnS_2_ nanosheets under light are depicted in Fig. 12. Under the illumination of solar simulator light, linear changes in photocurrent are observed in pristine SnS_2_ and Ni-SnS_2_ samples.

As the percentage of nickel loading increases the photocurrent also increases up to 2.5 mol% of nickel concentration and then it decreases. The 2.5 mol% Ni concentration of Ni-SnS_2_ shows the highest photocurrent almost 20 times enhanced than that of pristine SnS_2_. The result is clearly consistent with the enhancement in photocatalytic performance. Such behaviour can be attributed to the formation of Ni-SnS_2_ heterojunction, resulting in effective separation of electron–hole pairs, in-tern, allowing higher photoconductivity. Therefore, the enhanced photocatalytic activity is endorsed by the better photoconductivity of 2.5 mol% Ni loaded on SnS_2_ nanosheets. Overall, the study indicates that the Ni-SnS_2_ with 2.5 mol% concentration of nickel nanoparticles shows the highest photocurrent means that the generation of higher amounts of electron–hole pairs and its effective separation is due to Ni-SnS_2_ balance concentration upon illumination of light supporting higher photocatalytic performance. The surface area, H_2_ generation and the % apparent quantum efficiency (AQE) are tabulated in Table 1.

## 4 Conclusion

The Ni-SnS_2_ nanosheets with varying Ni concertation were synthesized. The formation of SnS_2_ hexagonal nanosheets was confirmed by FESEM analysis having a size of 300–500 nm diameter and decoration of nickel along with nickel oxide nanoparticles was confirmed by observation of FE-TEM. The photocatalytic activity of the resultant Ni-SnS_2_ catalyst was found to be higher than pure SnS_2_ nanosheets. The highest H_2_-evolution occurred when the usage of 2.5 mol% of Ni-SnS_2_ concentration. Thus, the presence of Ni on the surface of 2D SnS_2_ promotes the separation of electron–hole pairs and enhances photocatalytic activity. As the concentration of Ni increases more than 2.5 mol% on the surface of SnS_2_ the shading effect occurs and resulting the lowering of photocatalytic activity. In a nutshell, the improved photoconductivity due to Ni-loaded SnS_2_ resulting in efficient separation of electron–hole pair plays important role in governing the higher photocatalytic performance.

## Conflicts of interest

There is no conflict of interest.

## Supplementary Material

RA-013-D2RA07954B-s001
