# Supplementary material for: Ni loaded SnS2 hexagonal nanosheets for photocatalytic hydrogen generation via water splitting
Source: RSC Adv. 2023 Jan 16;13(4):2418–26. doi: 10.1039/d2ra07954b (PMC9841975; doi:10.1039/d2ra07954b)
Supplement: RA-013-D2RA07954B-s001 [file RA-013-D2RA07954B-s001.pdf]

Electronic Supplementary Material (ESI) for RSC Advances.
This journal is © The Royal Society of Chemistry 2023

Ni loaded SnS_2 Hexagonal Nanosheets for Photocatalytic Hydrogen Generation Via Water Splitting

Niteen Jawale, Sudhir Arbuj,* Govind Umarji, Manish Shinde, Bharat Kale, and Sunit Rane*

Materials for Renewable Energy and Sensor Division,
Centre for Materials for Electronics Technology, Off Pashan Road, Panchavati, Pune 411008,
Maharashtra, India.
E-mail: sudhir1305@gmail.com / sunit@cmet.gov.in
Tel:+912025899273: Fax:+912025898180

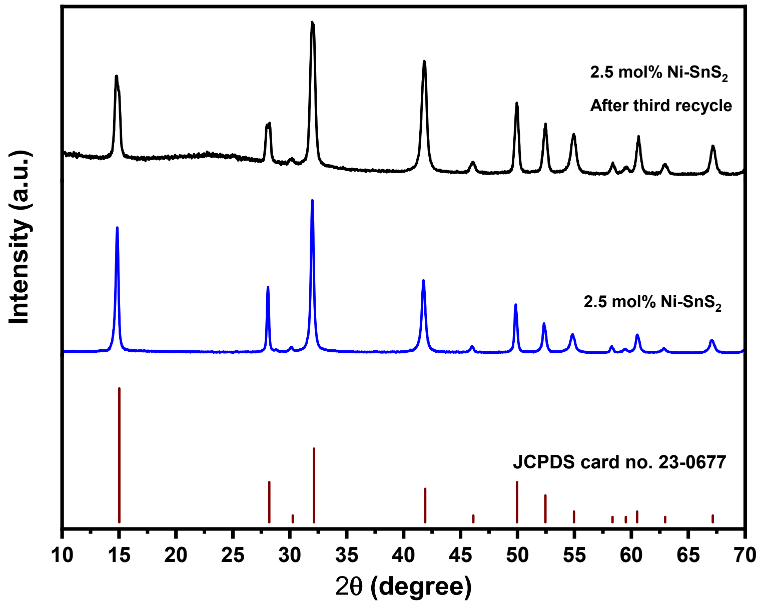

Figure S1. XRD analysis of Ni-SnS_2 nanostructures as synthesized (2.5mol% NI-SnS_2) and after water splitting reaction (2.5mol% Ni-SnS_2 third recycle).

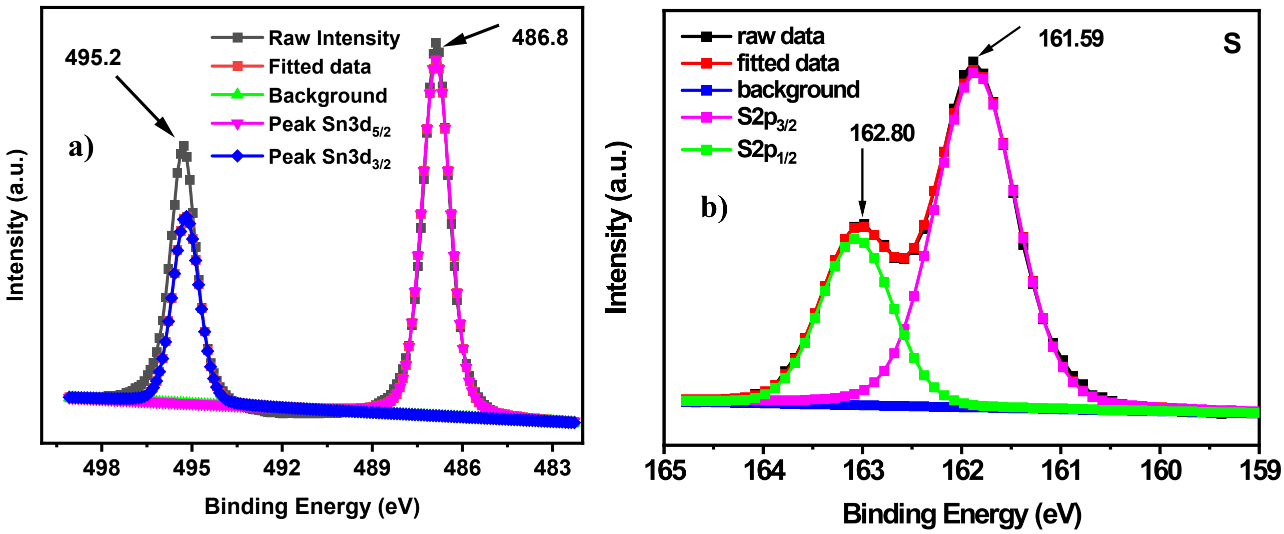

Figure S2. XPS analysis of pristine SnS_2 nanostructure a) for Sn and b) S.

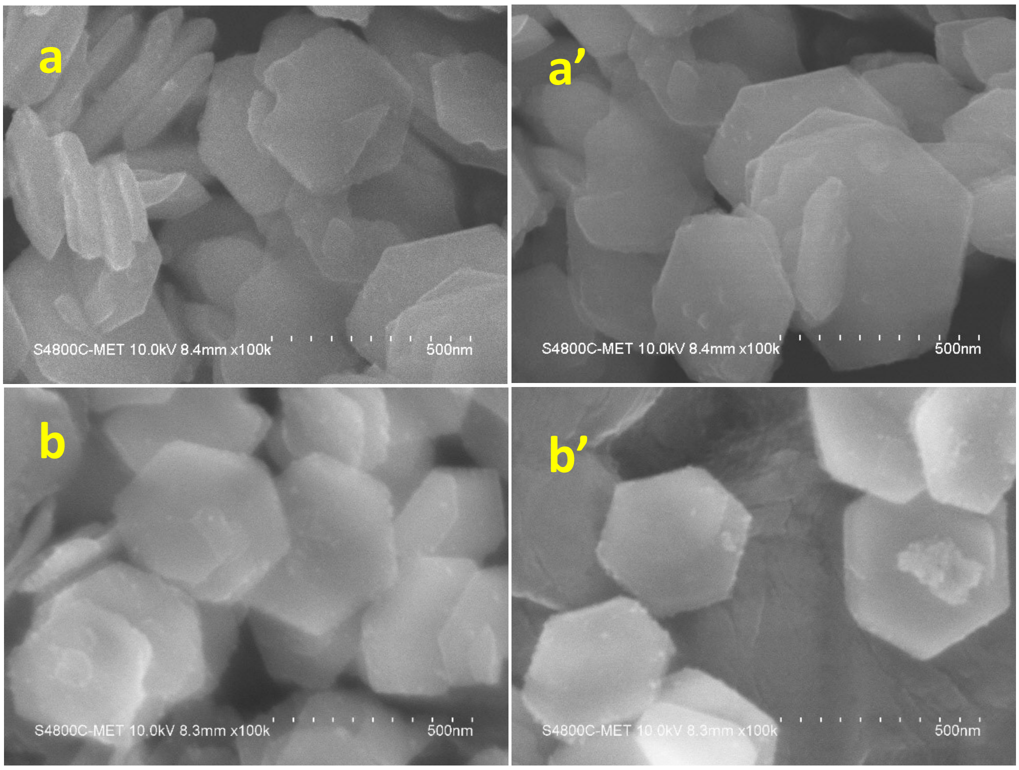

Figure S3. FE-SEM analysis of Ni-SnS_2 nanostructures, (a, a’) as synthesized, and recycled (b, b’) after water splitting reaction (third recycle).

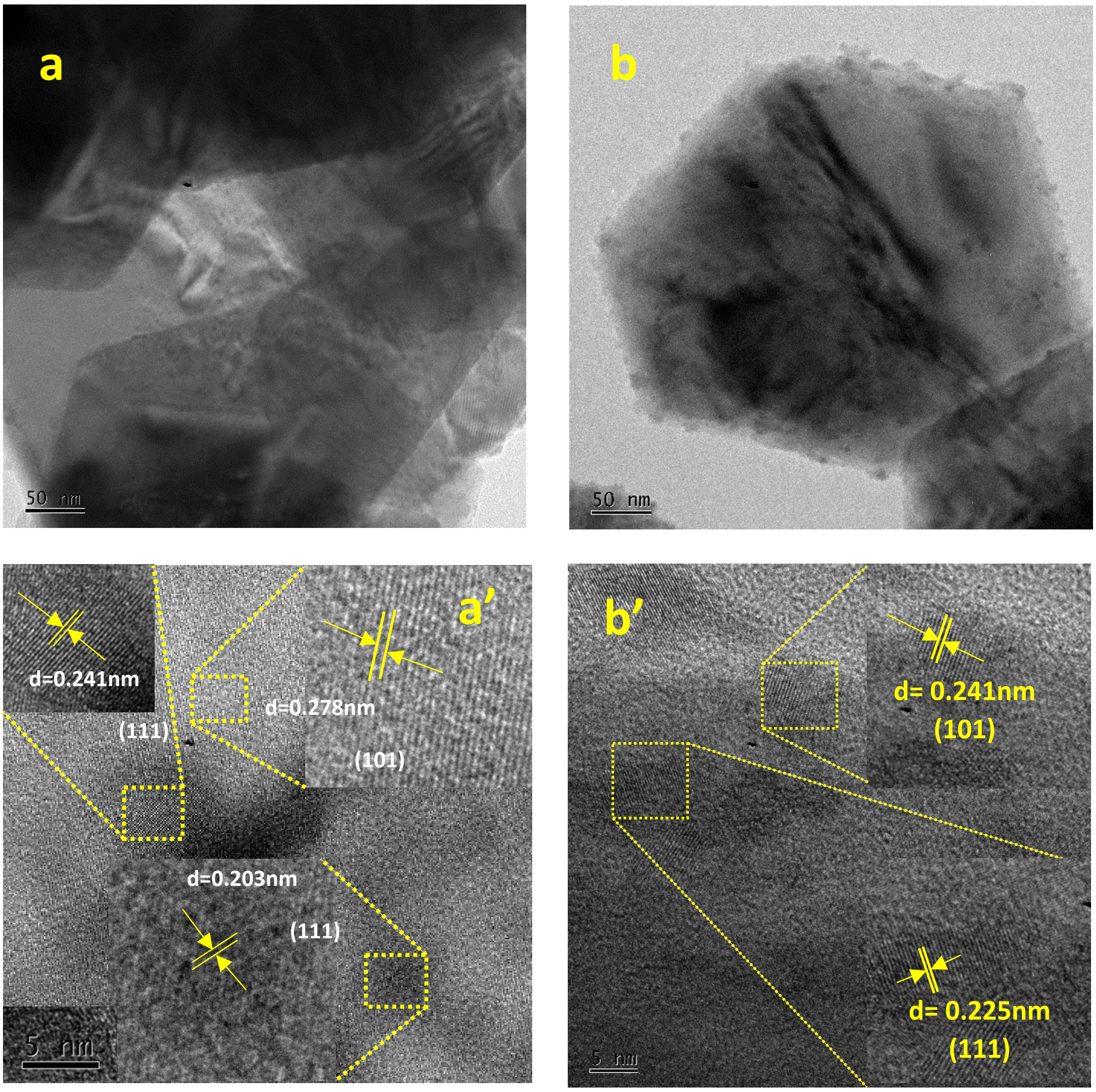

Figure S4. FETEM analysis of Ni-SnS_2, pristine 2.5mol% Ni-SnS_2 (a, a') and recycled 2.5mol% Ni-SnS_2 (b, b') images after water splitting reaction (third cycle).

(a)TEM image (a') HR-TEM image of as synthesized 2.5mol% Ni-SnS_2 respectively; (b) TEM image (b') HR-TEM image of 2.5 mol% Ni-SnS_2 nanosheet after water splitting reaction (third recycle) respectively. HR-TEM image (Fig. S4 a') exhibits the interplanar spacing of d=0.278nm corresponding to SnS_2 nanostructures with (101) plane, d=0.203 nm corresponding to Ni nanoparticle with (111) plane and d=0.241 corresponding to NiO nanoparticles with (111) plane, respectively. Further, HR-TEM image (S4b') of Pt-Ni-SnS_2 nanostructures shows lattice fringes with a spacing d= 0.241nm and d=0.225 nm corresponding to the (101) and (111) plane of NiO and cubic platinum nanoparticles respectively. It clearly indicates that the Ni-SnS_2 catalyst remain same even after third recycle.

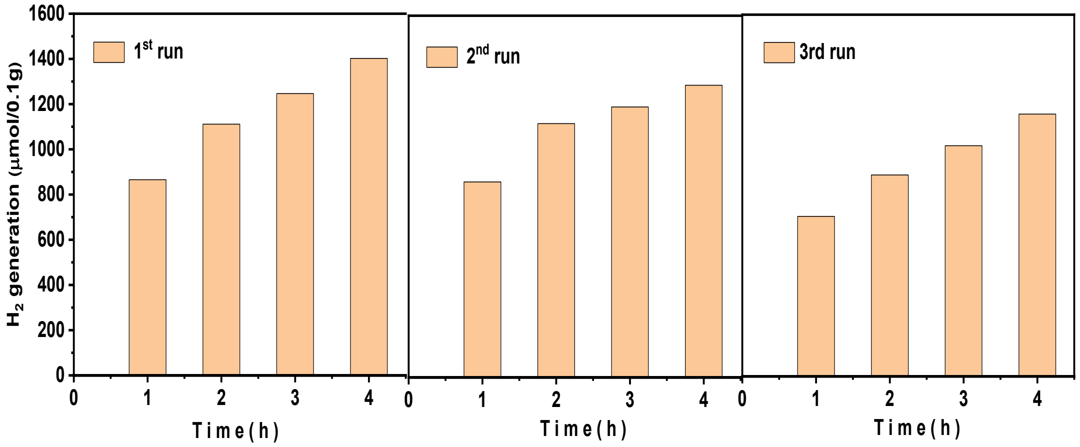

Figure S5: Recycling study of the 2.5mol% Ni-SnS_2 nanoplates for photocatalytic H_2-generation performance.

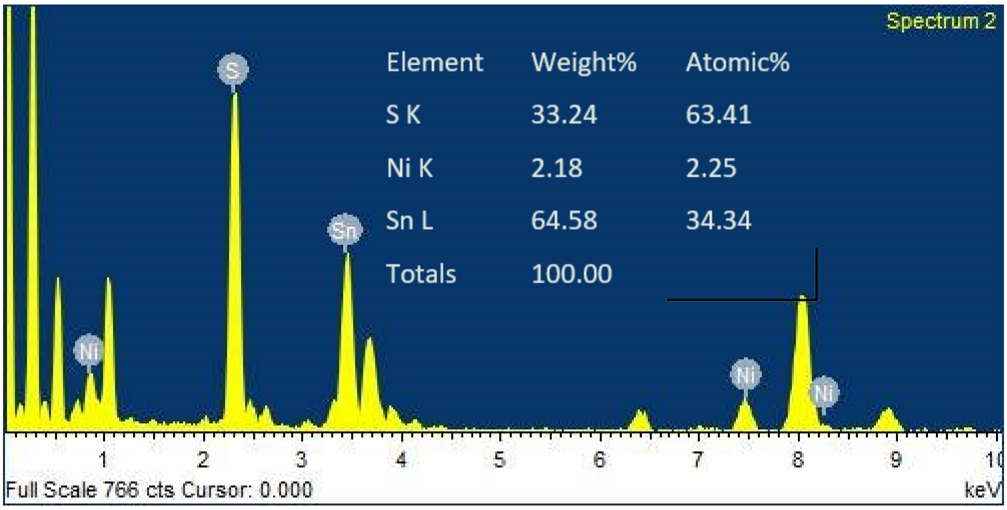

Fig S6: TEM-EDX spectra of 2.5 mol% Ni-SnS_2 nanostructure
